# The Antioxidative Fraction of White Mulberry Induces Apoptosis through Regulation of p53 and NFκB in EAC Cells

**DOI:** 10.1371/journal.pone.0167536

**Published:** 2016-12-09

**Authors:** AHM Khurshid Alam, ASM Sakhawat Hossain, Muhammad Ali Khan, Syed Rashel Kabir, Md Abu Reza, Md Mahbubur Rahman, Mohammad Saiful Islam, Md Aziz Abdur Rahman, Mamunur Rashid, Md Golam Sadik

**Affiliations:** 1 Department of Pharmacy, University of Rajshahi, Rajshahi, Bangladesh; 2 Department of Pharmacy, Bangabandhu Sheikh Mujibur Rahman Science and Technology University, Gopalganj, Bangladesh; 3 Department of Biochemistry and Molecular Biology, University of Rajshahi, Rajshahi, Bangladesh; 4 Department of Genetic Engineering & Biotechnology, University of Rajshahi, Rajshahi, Bangladesh; Emory University Winship Cancer Institute, UNITED STATES

## Abstract

In this study, the antioxidative fraction of white mulberry (*Morus alba*) was found to have an apotogenic effect on Ehrlich’s ascites carcinoma cell-induced mice (EAC mice) that correlate with upregulated p53 and downregulated NFκB signaling. The antioxidant activities and polyphenolic contents of various mulberry fractions were evaluated by spectrophotometry and the ethyl acetate fraction (EAF) was selected for further analysis. Strikingly, the EAF caused 70.20% tumor growth inhibition with S-phase cell cycle arrest, normalized blood parameters including red/white blood cell counts and suppressed the tumor weight of EAC mice compared with untreated controls. Fluorescence microscopy analysis of EAF-treated EAC cells revealed DNA fragmentation, cell shrinkage, and plasma membrane blebbing. These characteristic morphological features of apoptosis influenced us to further investigate pro- and anti-apoptotic signals in EAF-treated EAC mice. Interestingly, apoptosis correlated with the upregulation of p53 and its target genes PARP-1 and Bax, and also with the down-regulation of NFκB and its target genes Bcl-2 and Bcl-xL. Our results suggest that the tumor- suppressive effect of the antioxidative fraction of white mulberry is likely due to apoptosis mediated by p53 and NFκB signaling.

## Introduction

Apoptosis is the process of programmed cell death, which plays critical roles in a wide variety of physiological processes during fetal development as well as in adult tissues [1]. In most cases, apoptosis occurs through regulation of different types of pro-apoptotic (e.g. Bax, Bak, Bad) and anti-apoptotic (e.g. Bcl-2, Bcl-xL) genes [2]. Hanahan and Weinberg reported that the apoptotic trigger is initiated when pro- and anti-apoptotic genes are balanced, while imbalance allows proliferating cells to form cancer [3]. During cancer formation, expression of anti-apoptotic genes is upregulated, whereas pro-apoptotic genes are downregulated. Thus cancer cells escape apoptosis and its hallmarks, the distortion of cell morphology via chromatin condensation and nuclear fragmentation (pyknosis), plasma membrane blebbing, and cell shrinkage [4, 5]. Because cancerous cells are resistant to apoptosis, selective killing of these cells by promoting apoptosis is an effective goal for development of anti-cancer agents. Pro-apoptotic p53 signaling and anti-apoptotic NFκB signaling play critical roles in tumor development and progression, and are involved in angiogenesis, metastasis, and cell survival [6, 7, 8].

Researchers have proposed several hypotheses regarding causes of different types of cancers. Recently, the oxidative stress (OS) hypothesis revealed that OS is one of the important factors for development of different diseases, including cancers [9]. OS entails the imbalance between the production and scavenging of free radicals (e.g. H_2_O_2_, OH, etc), which are generated during normal physiological processes in the body. Free radicals can cause damage to cellular components, thereby producing cancer [10]. Wong and Goedde [11] reported that OS inhibits apoptosis through the induction of NFκB: this imbalanced condition can be ameliorated by antioxidants [12]. Saxena et al. [13] reported that antioxidants prevent cancer by promoting apoptosis. Moreover, Deepa et al. [14] reported that the antioxidant rich mulberry leaf extract induces apoptosis in human colon and breast cancers.

Plants are rich sources of bioactive compounds and offer drugs for the treatment of many diseases, including cancers [15]. Following a comprehensive literature review of medicinal plants available in Bangladesh, we chose to investigate white mulberry (*M*. *alba*). White mulberry is reported to have many promising biological activities including anti-inflammatory, anti-hepatoxic, anti-diabetic, anti-microbial, and immunomodulatory effects [16, 17, 18]. However, there is currently no data examining the antioxidant effect of white mulberry on molecular changes of genes involved in cancers.

Previously, mulberry extract was reported to possess *in vitro* cytotoxic [15], *in vivo* antioxidant [19], and anticancer activities [13, 14]. Recently, our group reported that the stem bark of white mulberry possessed stronger antioxidant properties than other parts, like leaves, fruits, and roots [20]. Therefore, the stem bark fraction was selected for further *in vitro* and *in vivo* analyses. In this study, we determined the apoptogenic effect of antioxidative white mulberry stem bark on tumorigenesis of EAC mice and find a correlation between antioxidant application, apoptosis, and changes in p53 and NFκB signaling pathways.

## Materials and Methods

### Collection of plant materials

Stem barks from white mulberry plants were collected from Rajshahi University Campus, Rajshahi, Bangladesh, in August, 2012. The plant was identified by an expert taxonomist at the Department of Botany, University of Rajshahi (voucher specimen no. 50). Plant materials (1 kg) were then washed with fresh DM water to remove dirty materials and shade dried for several days, with occasional sun drying. The dried materials were ground into coarse powder, passed through sieve #40 and stored at room temperature (RT) for future use.

### Extraction of plant materials

500 g of dried powdered plant materials were placed in an amber colored extraction bottle and soaked with 1.5 liters of methanol. The bottle and its contents were then sealed and kept for 15 days with occasional shaking and stirring. The whole mixture was filtered through cotton followed by Whatman No.1 filter papers, and was then concentrated with a rotary evaporator under reduced pressure at 40°C to afford a crude methanolic extract (CME) of 38 g of stem bark. The extract was then fractionated by pet-ether, chloroform, ethyl acetate, and finally with water to obtain petroleum ether (PEF, 7.08 gm), chloroform (CHF, 2.46 gm), ethyl acetate (EAF, 11.26 gm), and aqueous (AQF, 16.9 gm) fractions.

### Chemicals

1,1-diphenyl-2-picrylhydrazyl (DPPH), potassium ferricyanide, phosphate buffer, catechin (CA), ferrous ammonium sulphate, butylated hydroxytoluene (BHT), gallic acid (GA), ascorbic acid (AA), trichloro acetic acid (TCA), sodium phosphate, ammonium molybdate, DMSO, EDTA, thiobarbituric acid (TBA) and FeCl_3_ were purchased from Sigma Chemical Co. (St. Louis, MO,USA); vanillin was obtained from BDH; Folin-Ciocalteus's phenol reagent (FCR) and sodium carbonate were obtained from Merck (Damstadt, Germany). Ehrlich ascites carcinoma (EAC) cells were obtained with the courtesy of Department of Bio-Chemistry and Molecular Biology, Rajshahi University, Rajshahi, Bangladesh. TRIzole reagent, RNase OUT, dNTPs, Taq polymerase and SuperScript-III reverse transcriptase were purchased from Life Technologies (Invitrogen BioServices India Pvt. Ltd, Bangalore, India); Apo direct kit was purchased from BD Biosciences.

#### Estimation of total phenolics

Total phenolic contents were determined by the modified Folin-Ciocalteu method as described previously [21]. An aliquot of the extracts/a standard was mixed with 2 mL Folin-Ciocalteu reagent (previously diluted with water 1:10 v/v) and 2 mL (75 g/L) of sodium carbonate. The tubes were vortexed for 15 sec and allowed to stand for 20 min at 25°C for color development. Absorbance was then measured at 760 nm UV-spectrophotometer (Shimadzu, USA). Samples of extracts and standard were evaluated at a final concentration of 0.1 mg/mL. Total phenolic contents were expressed in terms of galic acid equivalent, GAE (standard curve equation: y = 0.096x+0.046, R^2^ = 0.999), mg of GA/g of dry extract.

#### Estimation of total flavonoids

Total flavonoids were estimated using the method as described previously [22]. To 0.5 ml of sample, 1.5 ml of methanol, 100 μl of 10% aluminum chloride, 100 μl of 1 M potassium acetate solution and 2.8 ml of distilled water was added. After 1.5 hours of incubation at RT, the absorbance was measured at 420 nm. Extract samples were evaluated at a final concentration of 0.1 mg/mL. Total flavonoids content was expressed in terms of catechin equivalent, CAE (standard curve equation: y = 0.0135x + 0.0085, R2 = 0.9984), mg of CA/g of dry extract.

#### Estimation of total flavonols

Total flavonol content in the plant extracts were estimated using the method as described previously [23]. To 2.0 mL of sample (standard), 2.0 mL of 2% AlCl3 in ethanol and 3.0 ml sodium acetate (50 g/L) solutions were added. The absorption at 440 nm was read after 2.5 hours at 20°C. Extract samples were evaluated at a final concentration of 0.1 mg/mL. Total flavonol content was expressed in terms of quercetin equivalent, QUE (standard curve equation: y = 0.0255x + 0.0069, R^2^ = 0.9995), mg of QU/g of dry extract.

#### Estimation of total proanthocyanidins

Determination of proanthocyanidins was based on a prior protocol [24]. A volume of 0.5 mL of 0.1 mg/mL extract solution was mixed with 3 mL of 4% vanillin-methanol solution and 1.5 ml hydrochloric acid. The mixture was allowed to stand for 15 minutes before absorbance was measured at 500 nm. Extract samples were evaluated at a final concentration of 0.1 mg/mL. Total content of proanthocyanidin was expressed in terms of catechin equivalent, CAE (standard curve equation: y = 0.567x − 0.024, R2 = 0.9801), mg of CA/g of dry extract.

#### Determination of total antioxidant capacity

Total antioxidant capacity (TAC) of samples/a standard was determined by a prior method [25]. 0.5 mL of samples/standard at different concentrations was mixed with 3 ml of reaction mixture containing 0.6 M sulphuric acid, 28 mM sodium phosphate and 1% ammonium molybdate and then incubated at 95°C for 10 min to complete the reaction. The absorbance was measured at 695 nm using a spectrophotometer against blank after cooling at RT. CA was used as standard. A typical blank solution contained 3 ml of reaction mixture and the appropriate volume of the same solvent used for the samples/standard were incubated at 95°C for 10 min and the absorbance was measured at 695 nm.

#### Determination of ferrous reducing antioxidant capacity

The ferrous reducing antioxidant capacity of samples/a standard was evaluated by a prior method [26]. A volume of 0.25 mL samples/standard of solution at different concentrations, 0.625 mL of potassium buffer (0.2 mol/L) and 0.625 mL of 1% potassium ferricyanide, K3[Fe(CN)6] solution were added into the test tubes. The reaction mixtures were incubated for 20 min at 50°C to complete the reaction. Then 0.625 mL of 10% TCA solution was added into the test tubes. The total mixture was centrifuged at 3000 r/min for 10 min. After which, 1.8 mL supernatant was withdrawn from the test tubes and was mixed with 1.8 mL of distilled water and 0.36 mL of 0.1% FeCl3 solution. The absorbance of the solution was measured at 700 nm using a spectrophotometer against blank. A typical blank solution contained the same solution mixture without plant extracts/standard was incubated under the same conditions and the absorbance of the blank solution was measured at 700 nm. Increased absorbance of the reaction mixture indicates increased reducing capacity.

#### Determination of DPPH radical scavenging assay

Free radical scavenging activity was determined by DPPH radical scavenging assay as described previously [27]. 2.4 mL of 0.1 mM DPPH in methanol was mixed with 1.6 ml of extracts at different concentrations. The reaction mixture was vortexed thoroughly and left in the dark at RT for 30 min. The absorbance of the mixture was measured by spectrophotometer at 517 nm. BHT was used as the reference standard. Percentage DPPH radical scavenging activity was calculated by the following equation:

$$\%\;DPPH\;radical\;scavenging\;activity=\{(A_{0}-A_{1})/A_{0}\}\times 100$$

where A_0_ is the absorbance of the control, and A_1_ is the absorbance of the extractives/standard. Percent of inhibition was plotted against concentration, and from the graph IC_50_ was calculated.

#### Determination of hydroxyl radical scavenging activity assay

Hydroxyl radical scavenging activity of the extracts/ a standard was measured according to prior protocol [28] with minor modification(s). The reaction mixtures contained 0.8 mL of phosphate buffer solution (50 mmol/L, pH 7.4), 0.2 mL of extracts/standard at different concentrations, 0.2 mL of ethylene diamine tetraacetic acid (1.04 mmol/L), 0.2 mL of FeCl3 (1 mmol/L) and 0.2 mL of 2-Deoxy-D-ribose (28 mmol/L) were taken in the test tubes. The mixtures were kept in a water bath at 37°C and the reaction was started by adding 0.2 mL of AA (2 mmol/L) and 0.2 mL of H_2_O_2_ (10 mmol/L). After incubation at 37°C for 1h, 1.5 mL of cold TBA (10 g/L) was added to the reaction mixture followed by 1.5 mL of HCl (25%). The mixture was heated at 100°C for 15 min and then cooled down with water. The absorbance of the solution was measured at 532 nm with a spectrophotometer. The hydroxyl radical scavenging activity was evaluated with the inhibition of percentage of 2-Deoxy-Dribose oxidation on hydroxyl radicals. The percentage of hydroxyl radical scavenging activity was calculated according to the following formula:

$$\%Hydroxyl\;radical\;scavenging\;activity=\{A_{0}-(A_{1}-A_{2}\}\times 100/A_{0}$$

where, A_0_ is the absorbance of the control without a sample, A_1_ is the absorbance after adding the sample and 2-Deoxy-D-ribose, A_2_ is the absorbance of the sample without 2-Deoxy-D-ribose. Then percentage of inhibition was plotted against concentration, and from the graph IC_50_ was calculated

#### Determination of Lipid peroxidation inhibition activity assay

The lipid peroxidation inhibition assay was determined according to prior method [29] with a slight modification. Excised rat liver was homogenized in buffer and then centrifuged to obtain liposome. 0.5 mL of supernatant, 100 μL 10 mM FeSO_4_, 100 μL 0.1 mM AA and 0.3 mL of extractives/standard at different concentrations were mixed to make the final volume 1 ml. The reaction mixture was incubated at 37°C for 20 min. One ml of (28%) TCA and 1.5 ml of (1%) TBA was added immediately after heating. Finally, the reaction mixture was again heated at 100°C for 15 min and cooled at RT. After cooling, the absorbance was taken at 532 nm. Percentage inhibition of lipid peroxidation was calculated by the following equation:

$$\%\;lipid\;peroxidation\;inhibition=\{(A_{0}-A_{1})/A_{0}\}\times 100$$

where A_0_ is the absorbance of the control, and A_1_ is the absorbance of the extractives/standard. Percent of inhibition was plotted against concentration, and IC_50_ was calculated from the graph.

#### Experimental animals

Swiss albino male mice, aged 4 weeks and weighing between 25–30 grams, were purchased from ICDDRB (International Centre for Diarrheal Disease Research in Bangladesh), Dhaka, Bangladesh. The animals were housed in propylene cages in a controlled environment (temperature 25±2°C and 12 h dark and light cycle) and received feed formulated by ICDDRB and water *ad libitum*. The animals were acclimatized to laboratory conditions for 10 days prior to initiation of experiments. To keep the hydration rate constant, food and water supply were stopped 12 hours before the experiments.

#### Ethical clearance

Protocol used in this study for the use of mice as an animal model for cancer research was approved by the Rajshahi University Animal Ethical committee (27/08/RUBCMB). This research work was approved by Ethical Review Committee of Research Cell of Rajshahi Medical College, Bangladesh (ref. RMC/ER/2010-2013/01).

#### Experimental design

Mice (12 mice per group) were randomly divided into four different groups: Group I, the normal group (non-tumor bearing) which received vehicle only; Group II, the EAC tumor-bearing untreated control group (which were intraperitoneally injected with 1×10^5^ exponentially grown EAC cells; Group III, the EAF-treated EAC tumor-bearing group; Group IV, the bleomycine-treated EAC tumor-bearing group.

#### Collection and administration of EAC cells

EAC cells were collected as described previously [4]. In brief, EAC cells were propagated intraperitoneally (biweekly) and the cells were collected from a donor Swiss albino mouse bearing 6–7 days old ascites tumors. The cells were adjusted to 1×10^5^ cells/mL via dilution with normal saline (0.9%) and counted by haemocytometer. The viability of tumor cells was observed by trypan blue (0.4%) exclusion assay.

#### Cell growth inhibition

*In vivo* tumor cell growth inhibition was carried out as described previously [30]. For this study, treatment was started after 24 hours of tumor inoculation and continued for 5 days. Group II mice were intraperitoneally injected with 1×10^5^ exponentially grown EAC cells. Group III received the test sample at 100 mg/kg per day, whereas Group IV received standard bleomycin at 0.3 mg/kg per day. In each case, the volume of the test solution injected (i.p.) was 0.1 mL/day per mouse. Out of 12 mice, four were sacrificed on the 6^th^ day after transplantation and tumor cells were collected by repeated i.p wash with 0.9% saline. The total number of tumor cells in the peritoneal cavity was counted by the trypan blue exclusion method using the Cedex cell counter (Roche). Viable tumor cells per mouse of the treated group were compared with those of the control (Group II). Cell growth inhibition was calculated by the following formula:

$$\%\;\mathrm{Cell}\;\mathrm{growth}\;\mathrm{inhibition}=(1-\frac{T_{w}}{C_{w}})\times 100)$$

Where, T_w_ = mean number of tumor cells of Group III or IV and C_w_ = mean number of tumor cells of Group II.

#### Haematological studies and tumor weight

The haematological parameters (WBC, RBC and Hb content) were determined by standard methods as described previously [31]. Blood was drawn from the tail of each group on the 12^th^ day of EAC-cell inoculation. Total WBC and RBC were counted by microscope with a haemocytometer, and the percentage of hemoglobin (Hb) was measured by hematometer. Tumor growth was monitored daily by measuring weight changes upto 20 days of treatment.

#### Observation of morphological change and nuclear damage

Cell apoptosis (nuclear condensation and fragmentation) was morphologically observed under a fluorescence microscope (OlympusiX71, Korea) using a prior method [4]. Hoechst 33342 staining was used to observe apoptotic morphology. In brief, EAC cells were collected from Group II and III and washed thrice with phosphate buffer saline (PBS). Cells were then stained with 0.1 gm/mL of Hoechst 33342 at 37^°^C for 20 min in a dark room, and were then washed again with PBS.

#### Detection of apoptosis by TUNNEL assay

The fragmented DNA of apoptotic cells was labeled by catalytically incorporating fluorescein-12-dUTP at the 3^/^-hydroxyl ends of the fragmented DNA using the enzyme terminal deoxynucleotidyl transferase (TdT) using Apo-direct kit. The cells were then analyzed on FACS (equipped with 488 nm Argon laser light source; 515 nm band pass filter, FL1-H, and 623 nm band pass filter, FL2-H) using CellQuest software (Becton Dickinson, San Jose, CA). Electronic compensation of the instrument was done to exclude overlapping of the emission spectra. A total of 10,000 events were acquired and dual parameter dot plot of FL2-H (xaxis; PI-fluorescence, linear scale) vs. FL1-H (y-axis; FITC-fluorescence, logarithmic scale) has been shown.

#### Cell cycle analysis

EAF treated (Group III) (100 mg/kg/day) and untreated (Group II) EAC cells were collected from mice after treatment of five days and washed thrice with cold PBS [32]. After fixation with 70% ethanol for 24 h at 4°C, the cells were washed thrice with cold PBS. Finally, the cells in 1 ml PBS were treated with 50 μl of RNase A (1 mg/ml) for 30 min at 37°C followed by staining with 5 μl of propidium iodide (1 mg/ml) in dark at 4°C for 5 min before analyzing using flowcytometry. The fractions of cells in G0/G1, S, and G2/M phase were analyzed by a FACS Flow cytometer (Partec CyFlow SL,Germany). Cell cycle phase distribution of nuclear DNA was determined on FACS, fluorescence detector equipped with 488 nm argon laser light source and 623 nm band pass filter (linear scale) using CellQuest software (Becton Dickinson). A total of 10000 events were acquired and analysis of data was performed using ModFit software. A histogram of DNA content (*x*-axis, PIfluorescence) versus counts (*y*-axis) has been displayed.

#### Total RNA isolation and cDNA preparation

Total RNA was extracted as described previously [33]. In brief, EAC cells (1 ×10^5^ cells/mL) were treated with 0.5 ml TRIzole reagent, vortexed gently for a few seconds and 0.2 ml CHCl_3_ was added followed by vigorous shaking. The reaction mixture was then incubated at RT for 2–3 min and the mixture was centrifuged at 12,000 rpm for 15 min at 4°C. The supernatant was transferred into a fresh eppendorf tube and then 500 μL of isopropranol was added and mixed properly. The reaction mixture was incubated at RT for 10 min and again centrifuged at 12,000 rpm for 10 min at 4°C. The supernatant was removed and an RNA pellet was observed at the bottom of the eppendorf tube. To obtain purified RNA, it was washed with 75% ethanol and centrifuged at 7,500 rpm at 4°C for 5 min. After removing the supernatant, the RNA pellet was dried at RT and dissolved with DEPC treated RNase free water. The concentration of RNA was measured with nanodrop (Thermo scientific nanodrop 8000).

For cDNA synthesis, 1 μg of purified total RNA was used in a total volume of 20 μL in the presence of 0.5 μg of oligo (dT)_**15**_ primer (Promega), 0.5 mM dNTPs, 1× first-strand buffer, 5 mM DTT (dithiothreitol), 2 units of RNase OUT (40 unit/μL) and 10 units of SuperScript III reverse transcriptase (200 unit/μL) (Invitrogen). The reaction was incubated for 1 hour at 50°C and then stopped by heating at 70°C for 15 min.

#### Reverse transcriptase polymerase chain reaction (RT- PCR)

To determine the expression levels of p53, Bax, PARP, Bcl-2 and NFκB, RT-PCR was performed as described previously [33]. In brief, the first-strand cDNA was amplified by PCR with specific primers. 25 μL reaction volumes were prepared containing 1X Taq polymerase buffer, 25 pmol each of forwarded and reverse primers, 2.5 mM of each dNTPs and 0.25 U of platinum Taq polymerase (Tiangen, China). The following specific oligonucleotides (IDT, Singapore) were used: Bcl-2 upstream-(5´-GTGGAGGAGCTCTTCAGGGA-3´) and Bcl-2 downstream-(5´AGGCACCCAGGGTGATGCAA-3´) generating a band of 0.304 kb; Bax upstream-(5´-GGCCCACCAGCTCTGAGCAGA-3´) and Bax downstream- (5´GCCACGTGGGCGTCCCAAAGT-3´) generating a band of 0.477 kb; Bcl-X upstream- (5´-TTGGACAATGGACTGGTTGA-3´) and Bcl-X downstream (5´GTAGAGTGGATGGTCAGTG-3´) generating two bands of 0.78 kb and 0.591 kb for Bcl-xL and Bcl-xS isoforms; PARP-1, upstream-(5´-AGGCCCTAAAGGCTCAGAAT-3´) and PARP-1 downstream-(5´-CTAGGTTTCTGTGTCTTGAC-3´) generating a band of 0.280 kb; GAPDH upstream-(5´-GTGGAAGGACTCATGACCACAG-3´) and GAPDH downstream-(5´-CTGGTGCTCAGTGTAGCCCAG-3´) generating a band of 0.475 kb; p53 upstream-(5´-GCGTCTTAGAGACAGTTGCCT-3´) and p53 downstream-(5´-GGATAGGTCGGCGGTTCATGC-3´) generating a band of 0.458 kb; NF´B upstream-(5´-AACAAAATGCCCCACGGTTA-3´) and downstream-(5´GGGACGATGCAATGGACTGT-3´) generating a band of 0.113 kb. The PCR products of these genes and GAPDH were electrophoresed in 1.5% agarose gel. The gels were stained with EtBr (ethidium bromide) and visualized in UV-trans illuminator (Vilber Lourmat).

### Statistical analysis

All analyses were carried out in triplicates. Data are presented as mean ± SD. The student’s unpaired t-test was used to evaluate significance between the test sample and control. One way analysis of variance (ANOVA) followed by a Dunnett Post hoc test was performed to evaluate significance differences among different groups. *P* value <0.05 was shown to be statistically significant. Free R-software version 2.15.1 (http://www.r-project.org/) and Microsoft Excel 2007 (Roselle, IL, USA) were used for the statistical and graphical evaluations.

## Results

### Determination of polyphenol contents

Polyphenols are the most abundant class of compounds in the plant kingdom and are important due to their antioxidant nature and various diseases curing abilities [34]. The white mulberry plant contains significant polyphenols. Table 1 shows the total polyphenol contents in the CME and its four fractions PEF, CHF, EAF and AQF. Of the fractions, EAF possessed the highest polyphenol content.

**Table 1:** Polyphenol content of CME, PEF, CHF, EAF and AQF.

Polyphenols	CME	PEF	CHF	EAF	AQF
Phenolics ^a^	35.93 ± 0.831	18.36 ± 0.48	46.55 ± 0.28	70.88 ± 0.17	22.71 ± 0.26
Flavonoids ^b^	102.46 ± 6.19	120.41 ± 0.266	44.28 ± 1.171	192.31 ± 0.504	14.65 ± 1.101
Flavonols ^c^	220.38 ± 1.26	179.25 ± 1.97	540.04 ± 1.42	736.77 ± 2.16	114.55 ± 1.18
Proanthocyanidins ^b^	4.68 ± 0.05	1.52 ± 0.04	3.83 ± 0.045	8.130 ± 0.088	1.01 ± 0.05

Each value is the average of three analyses ± standard deviation. a, b and c are expressed in terms of GAE, CAE and QUE, respectively (mg of GA, CA and QU/g of dry extract, respectively).

#### Determination of TAC

**Fig 1:**
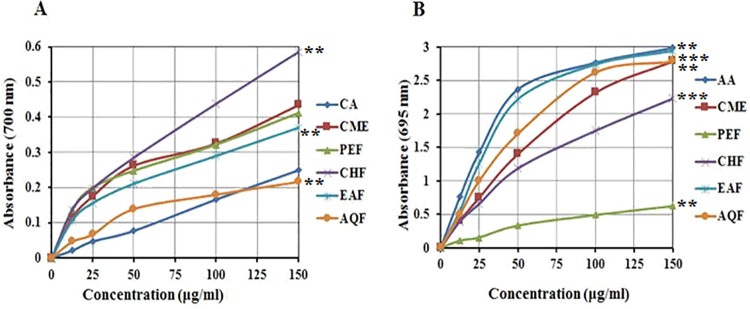
Determination of (A) total antioxidant capacity and (B) ferrous reducing antioxidant capacity of CME and its various fractions (PEF, CHF, EAF and AQF). Data expressed as mean ± SD (*n* = 3) for all tested dosages. Significant differences of EAF values are compared to values of standard and various fractions and marked as asterisk (*p < 0.05, **p < 0.01, and ***p < 0.001).

The total antioxidant potentials of white mulberry stem bark extracts were estimated from their ability to reduce the reduction of Mo (VI) to Mo (V) and the subsequent formation of a green phosphate/Mo (V) complex at an acidic pH. The TAC capacity of CME and its four fractions is shown in Fig 1A. The absorbance of CME, PEF, CHF, EAF, AQF and standard CA at 150 μg/mL were 0.434±0.008, 0.412±0.010, 0.585±0.009, 0.370±0.06, 0.216±0.007 and 0.249±0.004, respectively (Fig 1A). Significant (*p* < 0.01) antioxidant activity of EAF was detected compared to the other extractives and the CA control. EAF increased the total antioxidant activity with increasing concentration.

#### Determination of ferrous reducing antioxidant capacity

Reducing power is widely used to evaluate antioxidant activity of plant polyphenols. Reducing power is generally associated with the presence of reductones, which exert antioxidant action by breaking the free radical chains by donating a hydrogen atom. In this assay, the presence of reductants in the antioxidant sample causes the reduction of the Fe3+/ferricyanide complex to the Fe2+/ferrous form, and reducing power is monitored by measuring the formation of Perl’s Prussian blue at 700 nm [35]. In ferrous reducing antioxidant capability, the absorbance of CME, PEF, CHF, EAF, AQF and standard AA at 150 μg/ml were 2.79±0.007, 0.62±0.010, 2.23±0.010, 2.94±0.32, 2.79±0.12 and 2.99±0.107, respectively (Fig 1B). The reducing capacity of EAF is similar to that of standard AA and highest among all the tested extractives. The reducing activity increased with the increasing concentration of the extractives. We speculate that the reducing power of EAF is likely due to the presence of phenolic compounds, which could act as electron donors.

#### Determination of DPPH radical scavenging activity

**Fig 2:**
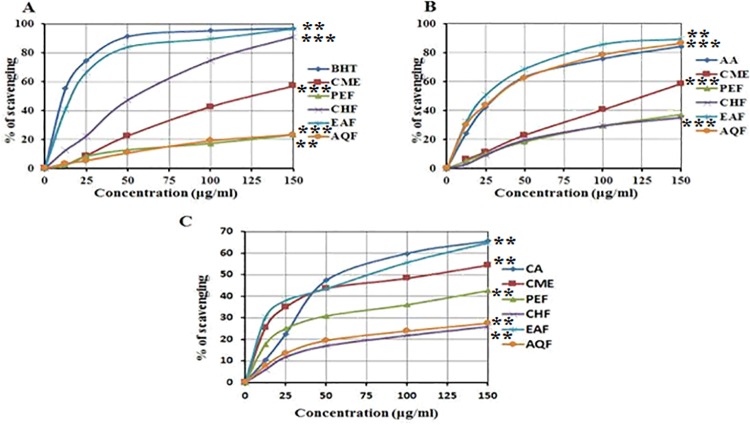
Determination of (A) DPPH radical scavenging activity, (B) OH radical scavenging activity, and (C) lipid peroxidation inhibition activity of CME and its various fractions (PEF, CHF, EAF and AQF). Data expressed as mean *±* SD (*n* = 3) for all tested dosages. Significant differences of EAF values are compared to values of standard and various fractions and marked as asterisk (*p < 0.05, **p < 0.01, and ***p < 0.001).

The effect of antioxidants on DPPH radicals is thought to be due to their hydrogen donating ability [36]. Radical scavenging activities are critical for preventing deleterious free radicals in different diseases, including cancers. DPPH free radical scavenging is an accepted mechanism by which antioxidants act to inhibit lipid peroxidation. This method has been used extensively to predict antioxidant activities because of the relatively short time required for analysis. The DPPH antioxidant capacity of CME, PEF, CHF, EAF, AQF and standard BHT was shown in Fig 2A. At 150 μg/mL, the DPPH free radical scavenging activity of CME, PEF, CHF, EAF, AQF and BHT were 56.95±0.53, 23.28±0.86, 90.99±0.44, 96.54±0.65, 23.47±0.51 and 96.88±0.23%, respectively (Fig 2A). Thus, EAF exhibited significant (*p* < 0.01) free radical scavenging activity when compared to control BHT and had a higher scavenging activity than all other extractives. It has been found that phenolics, flavonoids and tocopherols reduce the DPPH radicals by their hydrogen donating ability [37, 38]. The results obtained in this investigation reveal that EAF and all other fractions from white mulberry are free radical scavengers and able to react with DPPH radical.

#### Determination of hydroxyl radical scavenging activity

The mutagenic capacity of free radicals is due to the direct interactions of hydroxyl radicals with DNA [39]. Hydroxyl radicals can be generated by the biochemical conversion of the superoxide radical to superoxide dismutase by hydrogen peroxide, which subsequently produces extremely reactive hydroxyl radicals in the presence of divalent metal ions, such as iron and copper. The hydroxyl radicals scavenging activity of CME, PEF, CHF, EAF, AQF and standard AA at 150 μg /mL were 58.51±1.99, 37.21±1.6, 35.05±1.54, 89.40±2.63, 86.27±2.85 and 84.74±2.22%, respectively (Fig 2B). Our results reveal that EAF had a higher scavenging activity than that of the other extractives and even than that of the standard, AA. The ability of the extracts to quench hydroxyl radicals might directly be related to the prevention of lipid peroxidation.

#### Determination of lipid peroxidation inhibition activity assay

ROS induce membrane damage by peroxidising lipid moieties, especially the polyunsaturated fatty acids, in a chain reaction known as lipid peroxidation [40]. The initial reaction generates a second radical, which in turn can react with a second macromolecule to continue the chain reaction leads to cellular abnormalities. Lipid peroxidation is elevated in certain cancers [41]. The lipid peroxides scavenging activity of CME and its fractions was investigated and compared with the standard CA. At a concentration of 150 μg/mL, the scavenging activity of CME, PEF, CHF, EAF, AQF and standard were 54.36±1.23, 42.60±1.11, 25.84±0.61, 64.71±1.85, 27.44±0.61 and 65.54±2.27%, respectively (Fig 2C). The EAF exhibited higher activity than other extractives and showed similar activity to the standard CA. This result reveals that EAF differentially inhibits lipid peroxidation, likely by virtue of its free radical quenching potential. Thus, white mulberry is a good source for antioxidant and could be used as anticancer agent.

#### Effect of EAF on tumor cell growth

Antioxidants neutralize free radicals, which are a natural by-product of normal cell processes. In humans, the most common form of free radicals is oxygen. When an oxygen molecule (O2) becomes electrically charged or radicalized, it attempts to steal electrons from other molecules, causing damage to these molecules and DNA. Over time, such damage may become irreversible and lead to cancers. In this study, we evaluated the effect of EAF on EAC cell-induced tumorigenesis in mice and found significant (*p* < 0.01) inhibition of cell growth (70.20%) when compared to standard bleomycin (87.52%) (Table 2). The significant anti-tumor activity of EAF persuaded us to test whether its activity was due to the induction of apoptosis.

**Table 2:** Effect of EAF on EAC cell growth inhibition in mice (*in vivo*).

Name of Exp.	Nature of the	Dose	No. of EAC cells in mouse on	% of cell
	Drug	mg/kg/day (i.p)	day 6 after tumor cell inoculation	Growth inhibition
Control (EAC cell bearing mice)	-	-	(7.45±0.57)×106	-
Bleomycin	Standard	0.3 mg/kg	(0.62±0.05)× 107**	87.52
EAF	Experimental	100 mg/kg	(2.22±0.11)× 106**	70.20

Number of mice in each case (n = 6); the results are shown as mean ± SEM. Where significant value is **p<0.01

#### Effect of EAF on apoptosis

**Fig 3:**
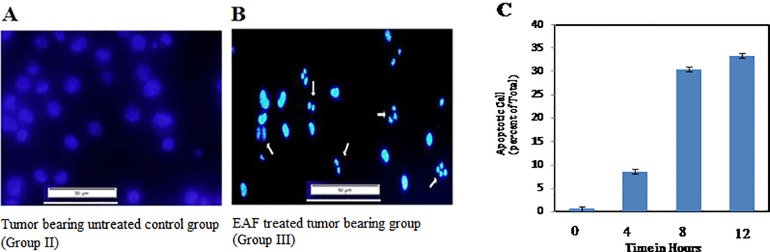
Determination of morphological changes of tumor bearing EAC cells and percentage of apoptosis by fluorescence microscopy and flowcytometry, respectively. (3A) Tumor-bearing untreated control group (Group II) versus (3B) EAF treated, tumor-bearing group (Group III); arrows indicate apoptotic features, including condensed chromatin, apoptotic bodies, plasma membrane blebbing, and nuclear fragmentation. (3C) EAC cells were treated with EAF *in vitro* for different time intervals and percentage of apoptotic cells of total cells was analyzed by flow cytometry. Data are shown as means of 3 independent experiments (n = 3).

Phenotypically, apoptosis is characterized by DNA fragmentation, cell shrinkage, chromatin compaction, plasma membrane blebbing, and collapse of the cell into small, intact fragments (apoptotic bodies). From our experiment, we observed that EAF treatment caused all of these phenotypes in EAC cells but not in untreated control cells (Fig 3A versus 3B), suggesting that EAF induces apoptosis in EAC cells. To confirm the nature of cell death, we further performed TUNNEL assay in which FITC-conjugated dUTP was incorporated into the DNA strand and breaks it due to apoptosis by terminal deoxynucleotidyl transferase. The flowcytometric analysis of the untreated EAC cells caused 96.40% viable cells with only 0.6% apoptotic cells. Interestingly, in comparison to untreated cells, EAF treated (150 μg/mL) showed significant increase in number of apoptotic cells (Fig 3C) in the range of 0.5–33.4%) in a time dependent manner indicating that EAF-induced tumor killing is likely due to apoptosis.

#### Effect of EAF on cell cycle arrest

**Fig 4:**
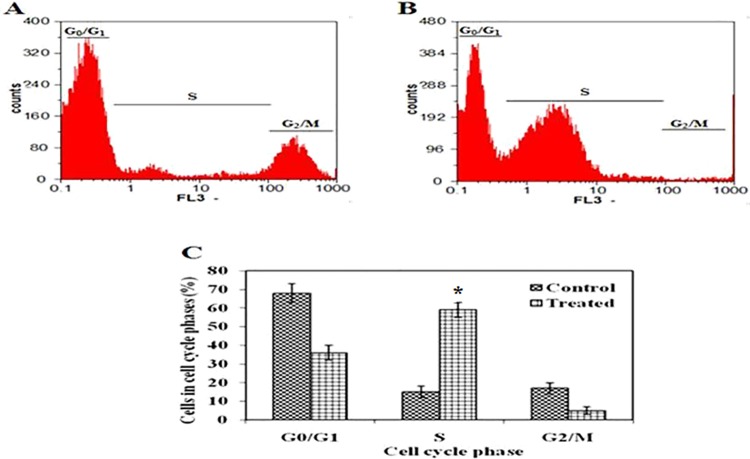
Effects of EAF on cell cycle distribution in EAC cells. The percentages of each cell cycle were evaluated by flow cytometry based on mean values obtained from two independent experiments. Results are expressed as mean ± S.D. as shown in (C). A and B represent flow cytometric analysis of untreated control and EAF treated EAC mice, respectively. The X-axis (FL3) represents the intensity of PI staining, which is directly proportional to the amount of DNA in cells, and theY-axis represents cell number. *p < 0.05 is EAF treated tumor bearing mice compared with untreated control group.

To further explore EAF’s tumor-killing mechanism, we exploited FACS and analyzed tumor cell cycle distributions (Fig 4A and 4B). The percentages of G0/G1, S, and G2/M phases in the untreated EAC cells was 68%, 16% and 18%, respectively (Fig 4C). After treatment with EAF, the S phase population increased to 58% and G0/G1 and G2/M phases decreased to 36% and 5%, respectively. These results suggest that the EAF significantly (*p* < 0.05) inhibits the cellular proliferation of EAC cells via S-phase arrest.

#### Effect of EAF on the expression of pro- and anti-apoptotic genes

**Fig 5:**
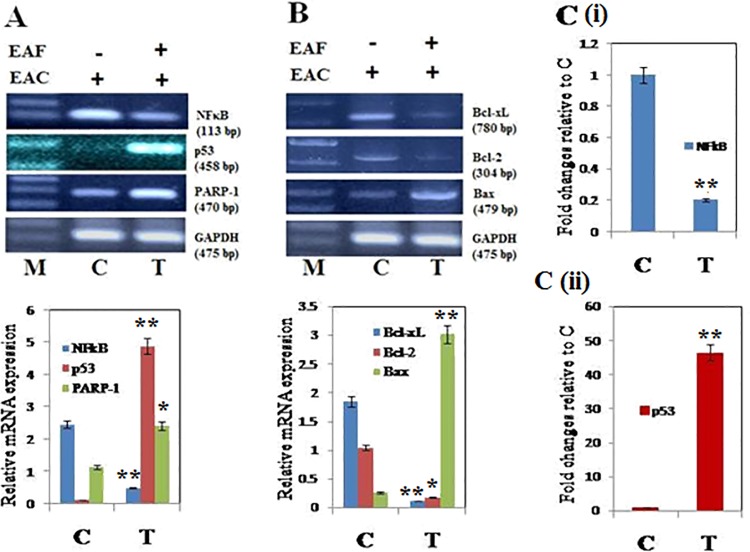
Analysis of mRNA of pro-and anti-apoptotic genes. Expression of (A, upper panel) NFκB, p53, and PARP-1 and (B, upper panel) Bcl-xL, Bcl-2, and Bax genes was analyzed by semi-quantitative RT-PCR in EAF-untreated EAC control mice (C) and EAF-treated EAC mice (T). The positions of the genes along with their length are indicated on the left in bp. The bottom panel shows the PCR products of GAPDH as a control. GAPDH transcript was used to normalize the expression levels. Relative expression of (A, lower panel) NFκB, p53, and PARP-1 and (B, lower panel) Bcl-xL, Bcl-2, and Bax genes was determined by a densitometric method. (C (i), upper and C (ii) lower panels) Fold changes of NFκB and p53 relative to untreated control (C) was determined by a densitometric method. Error bars indicate the S.D. from three different experiments. M represents 1 kb DNA ladder; C and T indicate control and EAF-treated mice, respectively. The asterisks indicate that EAF treated tumor bearing mice is significantly different (*p < 0.05, **p < 0.01, and ***p < 0.001) from untreated control group.

As it is well recognized that various genes play crucial roles in programmed cell death, we examined whether or not EAF affected the expression of pro-apoptotic genes like p53, PARP-1, and Bax and/or the expression of anti-apoptotic genes like NFκB, Bcl-2, and Bcl-xL. Using RT-PCR, we observed that in EAC cells, the mRNA levels of p53, PARP-1, and Bax increased (Fig 5A and 5B) while the mRNA levels of NFκB and its downstream target genes Bcl-2 and Bcl-xL decreased significantly (*p* <0.05) (Fig 5A and 5B). Thus, EAF treatment drives a decrease in the Bcl-2/Bax ratio. We also observed that p53 mRNA is 46-fold higher in EAF-treated mice when compared with controls, whereas NFκB mRNA is 5-fold lower in EAF-treated mice as compared to controls (Fig 5C). This analysis supports the model that EAF treatment shifts the balance of pro- and anti-apoptotic genes towards cell death.

#### Effect of EAF on haematological parameters and tumor weight

**Fig 6:**
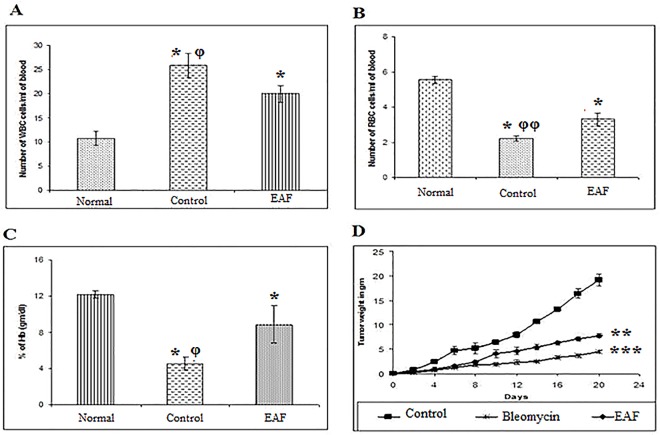
Differences in the hematological parameters of normal group (Group I: non tumor-bearing, which received vehicle only), untreated tumor-bearing control mice (Group II), and EAF-treated tumor-bearing mice (Group III) on day 12 after tumor inoculation. (A) White blood cell (WBC) count, (B) Red blood cell (RBC) count, (C) % of Hemoglobin (Hb). Significant differences of Group II and Group III was compared with Group I marked as asterisk and phi represents significant differences between Group III and Group II (*/^φ^*p* < 0.05, **/ ^φ φ^*p* < 0.01, and ***/ ^φφφ^*p <* 0.001). Tumor weight differences (D) was measured and % of tumor weight was calculated compared to Group II, Group III, and Group IV (bleomycin-treated tumor-bearing mice) daily per mouse upto 20 days of treatment. Data are representative of three independent experiments (4 mice per group). Significant differences of Group III and Group IV was compared with Group II marked as asterisk (*p < 0.05, **p < 0.01, and ***p < 0.001).

The effects of EAF on haematological parameters are shown in Fig 6A–6C. In EAC mice, WBC, RBC, and hemoglobin were significantly disrupted as compared to controls. Notably, these parameters reverted to normal when treated with EAF at a dose of 100 mg/kg (i.p.) (Fig 6A–6C). Moreover, the tumor burden was also reduced when treated with EAF and standard bleomycin (Fig 6D). Tumor weight was increase by 85.5% for Group II mice (EAC tumor-bearing untreated control mice). Interestingly, treatment of EAF and bleomycin significantly (*p* <0.05) reduced tumor weight of 21% for Group III mice (EAF-treated EAC tumor-bearing mice) and 27% for Group IV mice (bleomycin-treated EAC tumor-bearing mice), respectively after 20 days of treatment.

## Discussion

Numerous scientific reviews and experimental studies have described a relationship between increased cellular ROS and the pathogenesis of chronic diseases, including cancers [42, 43]. Hence, prevention of oxidative damage caused by ROS is an important strategy for the prevention and treatment of cancer. Plants rich in antioxidative polyphenols have been linked to a decreased risk for developing cancer through various mechanisms, including apoptosis [44, 45]. Thus, intensive efforts have been undertaken to search for plant-derived antioxidants and anti-cancer agents that are both effective and safe [45, 46].

Antioxidants can prevent and stabilize the cellular damage caused by cancer. Recent biological studies suggest that antioxidant rich plants induce apoptosis in many types of cancers (eg. colon, breast, liver) [13, 14]. In our previous work [20], we showed that the highest antioxidant property of white mulberry is found in the stem bark. The present study is a continuation of our previous finding to determine whether or not this antioxidative fraction possesses anti-cancer properties through the regulation of genes involved in apoptosis.

For assessment of antioxidant and free radical scavenging potential of white mulberry stem bark, several *in vitro* assay techniques were used, including total polyphenolic content, free radical scavenging, and antioxidant activity assays. From these data, we found that the ethyl acetate fraction (EAF) of white mulberry stem bark possesses the highest antioxidant activity among all other fractions. Thus, following the previous anti-proliferative activity reports of mulberry [15, 47], we set out to determine the apoptogenic effect of EAF on tumorigenesis in EAC mice.

Our evidences from chemical, cell culture, and animal studies indicates that antioxidants may slow or possibly prevent the development of cancer [48]. In this study, we demonstrated that treatment of EAC mice with white mulberry EAF significantly suppresses tumor cell growth *in vivo* (Table 2). The anti-tumor activity of white mulberry was similar to other natural products like black tea, honey and jacalin, against EAC tumorigenesis [49, 50, 51]. We also demonstrated that EAF treatment triggered morphological changes of EAC cells, including DNA fragmentation, cell shrinkage, and DNA condensation (Fig 3), which are characteristic features of apoptosis [5]. These morphological changes were observed clearly when compared with untreated EAC cells (Fig 3A and 3B) and are consistent with recent reports [4, 5]. Moreover, we also investigated DNA fragmentation by TUNNEL assay to confirm the nature of apoptosis and observed that EAF treatment significantly increases the percentage of apoptotic cell when compared with control (Fig 3C). Additionally, EAF treatment normalized the blood parameters of EAC mice when compared to untreated mice (Fig 6), suggesting that EAF could be considered for cancer-associated blood disorders. Together, these results reveal that white mulberry is important for inhibiting tumor cell growth and for inducing apoptosis.

Many anti-tumor and DNA-damaging agents induce apoptosis by arresting the cell cycle at the G1, S, or G2/M phases [52]. For example, treatment with quercetin, one of the most abundant flavonoids found in fruits and vegetables, lead to various cell cycle arrests in leukemia, colorectal carcinoma, breast carcinoma, and oesophageal adenocarcinoma cell lines [53]. We therefore checked whether EAF-induced apoptosis altered the tumor cell cycle and found that EAF-induced cell cycle arrest in S-phase (Fig 4). This suggests that the apoptogenic effect of EAF is mediated by S-phase arrest [54].

Cancers have various strategies to escape death, including altered expression of genes and proteins involved in cell survival [55]. One common survival strategy is to escape from apoptosis by deregulation of pro-apoptotic genes [56] or hyper-activation of anti-apoptotic genes [57]. Therefore, cancer specific induction of apoptosis is thought to be a good strategy for cancer treatment.

It is well recognized that whether a cell becomes committed to apoptosis partly depends upon the balance between proteins that mediate cell cycle arrest and cell death (e.g. p53, PARP-1, Bax) and proteins that promote cell viability (e.g. Bcl-2 and Bcl-xL) [58, 59]. p53 is the a key modulator of apoptosis following DNA damage and cell cycle arrest [60] and binds Bcl-2 family members, thus freeing Bax to transmit an apoptotic signal to the mitochondria that ultimately leads to cell death [61]. The upregulation of p53 is also responsible for the upregulation of the pro-apoptotic PARP-1 gene [62]. In this study, our findings (Fig 5) suggested that upregulation of p53 led to subsequent binding and downregulation of Bcl-2 and Bcl-xL expression. Downregulation of Bcl-2 and Bcl-xL increases PARP-1 and Bax, thereby decreasing the Bcl-2/Bax ratio and promoting apoptosis and death. Accordingly, we observed a decrease in NFκB signaling, which inhibits apoptosis by Bcl-2 and Bcl-xL [63]. Thus, the anti-tumor effect of EAF is linked with the inhibition of NFκB and an increase in p53 signaling, consistent with prior findings [63]. Taken together, these data support the idea that treatment of antioxidant-rich white mulberry stem bark induces apoptosis and inhibits tumor cell growth by promoting S-phase cell cycle arrest and shifting the balance between pro-and anti-apoptotic signaling pathways.

## Conclusion

In this study, we found that the antioxidant rich fraction of white mulberry stem bark inhibits the growth of Ehrlich ascites carcinoma cells more effectively. We uncovered that these apoptogenic effects appear to be mediated by multiple mechanisms, including inhibition of ROS, deregulation of cell cycle and S-phase arrest, and the induction of apoptosis through the regulation of target genes, p53 and NFκB. Further study is required to clarify the relationship and exact molecular mechanisms linking antioxidants with cancer.

## References

[1] Elmore S. Apoptosis: a review of programmed cell death. Toxicol Patho. 2007;35(4):495–516. 10.1080/01926230701320337 PMC2117903 17562483

[2] Reed JC. Mechanism of apoptosis. Am J Pathol. 2000;157(5):1415–1430. 10.1016/S0002-9440(10)64779-7 11073801 PMC1885741

[3] Hanahan D, Weinberg RA. Hallmarks of cancer: the next generation. Cell. 2011;144(5):646–674. 10.1016/j.cell.2011.02.013 21376230

[4] Kabir SR, Nabi MM, Haque A, Zaman RU, Mahmud ZH, Reza MA. Pea lectin inhibits growth of ehrlich ascites carcinoma cells by inducing apoptosis and G2/M cell cycle arrest in vivo in mice. Phytomedicine. 2013;20(14):1288–1296. 10.1016/j.phymed.2013.06.010 23867650

[5] Kerr JF, Wyllie AH, Currie AR. Apoptosis: a basic biological phenomenon with wide-ranging implications in tissue kinetics. Br J Cancer. 1972;26(4):239–257. 4561027 10.1038/bjc.1972.33 PMC2008650

[6] Karin M, Cao Y, Greten FR, Li ZW. NFκB in cancer: from innocent bystander to major culprit. Nat Rev Cancer. 2002;2(4):301–310. 10.1038/nrc780 12001991

[7] Garg A, Aggarwal BB. Nuclear transcription factor-kappa B as a target for cancer drug development. Leukemia. 2002;16(6):1053–1068. 10.1038/sj.leu.2402482 12040437

[8] Jochum W, Passegue E, Wagner EF. AP-1 in mouse development and tumorigenesis. Oncogene. 2001;20(19):2401–2412. 10.1038/sj.onc.1204389 11402336

[9] Lopez-Lazaro M. A new view of carcinogenesis and an alternative approach to cancer therapy. Mol Med. 2010;16(3–4):144–153. 10.2119/molmed.2009.00162 20062820 PMC2802554

[10] Halliwell B. Oxidative stress and cancer: have we moved forward? Biochem J. 2007;401(1):1–11. 10.1042/BJ20061131 17150040

[11] Wong GH, Goeddel DV. Induction of manganous superoxide dismutase by tumor necrosis factor: possible protective mechanism. Science. 1988;242(4880):941–944. 3263703 10.1126/science.3263703

[12] Arfan M, Khan R, Rybarczyk A, Amarowicz R. Antioxidant activity of mulberry fruit extracts. Int J Mol Sci. 2012;13(2):2472–2480. 10.3390/ijms13022472 22408465 PMC3292034

[13] Saxena A, Saxena AK, Singh J, Bhushan S. Natural antioxidants synergistically enhance the anticancer potential of AP9-cd, a novel lignan composition from Cedrus deodara in human leukemia HL-60 cells. Chem Biol Interact. 2010;188(3):580–590. 10.1016/j.cbi.2010.09.029 20932957

[14] Deepa M, Sureshkumar T, Satheeshkumar PK, Priya S. Antioxidant rich Morus alba leaf extract induces apoptosis in human colon and breast cancer cells by the downregulation of nitric oxide produced by inducible nitric oxide synthase. Nutr Cancer. 2013;65(2):305–310. 10.1080/01635581.2013.748924 23441618

[15] Naowaratwattana W, De-Eknamkul W, De Mejia EG. Phenolic-containing organic extracts of mulberry (Morus alba L.) leaves inhibit HepG2 hepatoma cells through G2/M phase arrest, induction of apoptosis and inhibition of topoisomerase IIα activity. J Med Food. 2010;13(5):1045–1056. 10.1089/jmf.2010.1021 20828312

[16] Balasubramanian A, Ramalingam K, Krishnan S, Ajm C. Anti-inflammatory activity of Morus indica Linn. Iranian J Pharmacol Ther. 2004;4(1):13–15.

[17] Hussein MS, El-Tawil OS, Yassin NEH, Abdou KA. The protective effect of Morus alba and Calendula officinalis plant extracts on carbon tetrachloride-induced hepatotoxicity in isolated rat hepatocyte. J Am Sci. 2010; 6(10):762–773.

[18] Khalid N, Fawad SA, Ahmed I. Antimicrobial activity, phytochemical profile and trace minerals of black mulberry (Morus nigra l.) fresh juice. Pak. J. Bot. 2011;43: 91–96.

[19] Wang Y, Xiang L, Wang C, Tang C, He X. Antidiabetic and antioxidant effects and phytochemicals of mulberry fruit (*Morus alba* L.) polyphenol enhanced extract. PLoS One. 2013;8(7):e71144 10.1371/journal.pone.0071144 23936259 PMC3728024

[20] Khan MA, Rahman AA, Islam S, Khandokhar P, Parvin S, Islam MB, et al A comparative study on the antioxidant activity of methanolic extracts from different parts of *Morus alba* L. (Moraceae). BMC Res Notes. 2013;6:24 10.1186/1756-0500-6-24 23331970 PMC3559264

[21] Wolfe K, Wu X, Liu RH. Antioxidant activity of apple peels. J Agric Food Chem. 2003;51(3):609–614. 10.1021/jf020782a 12537430

[22] Ordon-ez AAL, Gomez JD, Vattuone MA, Isla MI. Antioxidant activities of *Sechium edule* (Jacq.) swart extracts. Food Chem. 2006; 97(3):452–458.

[23] Kumaran A, Karunakaran RJ. In vitro antioxidant activities of methanol extracts of five *Phyllanthus* species from India. Lebenson Wiss Technol. 2007;40(2):344–352.

[24] Sun JS, Tsuang YH, Chen IJ, Huang WC, Hang YS, Lu FJ. An ultra-weak chemiluminescence study on oxidative stress in rabbits following acute thermal injury. Burns. 1998;24(3):225–231. 9677025 10.1016/s0305-4179(97)00115-0

[25] Prieto P, Pineda M, Aguilar M. Spectrophotometric quantitation of antioxidant capacity through the formation of a phosphomolybdenum complex: specific application to the determination of vitamin E. Anal Biochem. 1999;269(2):337–341. 10.1006/abio.1999.4019 10222007

[26] Jayanthi P, Lalitha P. Reducing power of the solvent extracts of Eichhornia crassipes (Mart.) Solms. Int J Pharm Pharm Sci. 2011;3(3):126–128.

[27] Blois MS. Antioxidant determinations by the use of a stable free radical. Nature. 1958;181:1199–1200.

[28] Nagai T, Myoda T, Nagashima T. Antioxidative activities of water extract and ethanol extract from field horsetail (tsukushi) Equisetum arvense L. Food Chem. 2005;91:389–94.

[29] Liu F, Ng TB. Antioxidative and free radical scavenging activities of selected medicinal herbs. Life Sciences, 2000;66(8):725–735. 10680580 10.1016/s0024-3205(99)00643-8

[30] Sur P, Ganguly DK. Tea plant root extract (TRE) as an antineoplastic agent. Planta Med. 1994;60(2):106–109. 10.1055/s-2006-959427 8202557

[31] Khanam JA, Islam MF, Jesmin M, Ali MM. Antineoplastic activity of acetone semicarbazone (ASC) against ehrlich ascites carcinoma (EAC) bearing mice. J Natl Sci Found. 2010;38(4):225–231.

[32] Krishan A. Rapid flow cytofluorometric analysis of mammalian cell cycle by propidium iodide staining. J Cell Biol. 1975;66(1):188–193. 49354 10.1083/jcb.66.1.188 PMC2109516

[33] Alam AH, Suzuki H, Tsukahara T. Retinoic acid treatment and cell aggregation independently regulate alternative splicing in P19 cells during neural differentiation. Cell Biol Int. 2010;34(6):631–643. 10.1042/CBI20090332 20230377

[34] Landete JM. Dietary intake of natural antioxidants: vitamins and polyphenols. Crit Rev Food Sci Nutr. 2013; 53(7): 706–721 10.1080/10408398.2011.555018 23638931

[35] Oktay M, Gülçin I, Küfrevioglu OI. Determination of in vitro antioxidant activity of fennel (Foeniculum vulgare) seed extracts. LWT-Food Sci Technol. 2003;36:263–271

[36] Choi HY, Jhun EJ, Lim BO. Application of flow injectionchemilumineacence to the study of radical scavenging activity in plants. Phytother Res. 2000; 14:250–253 10861967 10.1002/1099-1573(200006)14:4<250::aid-ptr587>3.0.co;2-j

[37] Duan X, Wu G, Jiang Y. Evaluation of the antioxidant properties of litchi fruit phenolics in relation to pericarp browning prevention. Molecules. 2007;12(4):759–771. 17851428 10.3390/12040759 PMC6149334

[38] Li H, Wang X, Li Y, Li P, Wang H. Polyphenolic compounds and antioxidant properties of selected China wines. Food Chem. 2009; 112(2):454–460.

[39] Baumann J, Wurn G, Bruchlausen FV. Prostaglandin synthetase inhibiting O-2 radical scavenging properties of some flavonoids and related phenolic compounds. Naunyn Schmiedebergs Arch Pharmacol. 1979; 308:27–32

[40] Scully C: Oral cancer: New insight into pathogenesis. Dent Update. 1993;20(3):95–100. 8224347

[41] Klaunig JE, Xu Y, Isenberg JS, Bachowski S, Kolaja KL, Jiang J, et al The role of oxidative stress in chemical carcinogenesis. Environ Health Perspect. 1998;106 (1):289–295. 9539021 10.1289/ehp.98106s1289 PMC1533298

[42] Durackova Z. Some current insights into oxidative stress. Physiol Res. 2010;59(4):459–469. 19929132 10.33549/physiolres.931844

[43] Visconti R, Grieco D. New insights on oxidative stress in cancer. Curr Opin Drug Discov Devel. 2009; 12(2):240–250. 19333869

[44] Islam S, Nasrin S, Khan MA, Hossain ASM, Islam F, Khandokhar P, et al Evaluation of antioxidant and anticancer properties of methanolic extract and its various fractions from the seeds of *Syzygium fruticosum* Roxb. (Myrtaceae) growing in Rajshahi, Bangladesh. BMC Complement Altern Med. 2013; 13:142 10.1186/1472-6882-13-142 23800021 PMC3691922

[45] Kwon HK, Hwang JS, So JS, Lee CG, Sahoo A, Ryu JH, et al Cinnamon extract induces tumor cell death through inhibition of NFkappaB and AP1. BMC Cancer. 2010;10:392 10.1186/1471-2407-10-392 20653974 PMC2920880

[46] Nomura M, Ma W, Chen N, Bode AM, Dong Z. Inhibition of 12-O tetradecanoylphorbol-13-acetate-induced NF-kB activation by tea polyphenols, (–)-epigallocatechin gallate and theaflavins. Carcinogenesis. 2000; 21(10):1885–1890. 11023547 10.1093/carcin/21.10.1885

[47] Chon SU, Kim YM, Park YJ, Heo BG, Park YS, Gorinstein S. Antioxidant and antiproliferative effects of methanol extracts from raw and fermented parts of mulberry plant (Morus alba L.). Euro Food Res and Tech. 2009;230(2):231–237

[48] Blot WJ, Li JY, Taylor PR, Guo W, Dawsey S, Wang GQ, et al Nutrition intervention trials in Linxian, China: supplementation with specific vitamin/mineral combinations, cancer incidence, and disease-specific mortality in the general population. J Natl Cancer Inst. 1993, 85:1483–1491. 8360931 10.1093/jnci/85.18.1483

[49] Bhattacharyya A, Choudhuri T, Pal S, Chattopadhyay S, K Datta G, Sa G, et al Apoptogenic effects of black tea on Ehrlich’s ascites carcinoma cell. Carcinogenesis. 2003;24(1):75–80. 12538351 10.1093/carcin/24.1.75

[50] Jaganathan SK, Mondhe D, Wani ZA, Pal HC, Mandal M. Effect of honey and eugenol on Ehrlich Ascites and solid carcinoma. J Biomed Biotechnol. 2010;2010:989163 10.1155/2010/989163 20369055 PMC2846731

[51] Ahmed H, Chatterjee BP, Debnath AK. Interaction and in vivo growth inhibition of Ehrlich ascites tumor cells by jacalin. J Biosci. 1988;13(4):419–424

[52] Gomes A, Giri B, Alam A, Mukherjee S, Bhattacharjee P, Gomes A. Anticancer activity of a low immunogenic protein toxin (BMP1) from Indian toad (Bufo melanostictus, Schneider) skin extract. Toxicon. 2011;58(1):85–92. 10.1016/j.toxicon.2011.05.008 21635912

[53] Srivastava S, Somasagara RR, Hegde M, Nishana M, Tadi SK, Srivastava M, et al Quercetin, a Natural Flavonoid Interacts with DNA, Arrests Cell Cycle and Causes Tumor Regression by Activating Mitochondrial Pathway of Apoptosis. Sci Rep. 2016; 6:24049 10.1038/srep24049 27068577 PMC4828642

[54] Zhang Y, Rishi AK, Dawson MI, Tschang R, Farhana L, Boyanapalli M, et al S-phase arrest and apoptosis induced in normal mammary epithelial cells by a novel retinoid. Cancer Res. 2000;60(7):2025–2032. 10766194

[55] Croce CM. Oncogenes and cancer. N Engl J Med. 2008;358:502–511. 10.1056/NEJMra072367 18234754

[56] Fesik SW. Promoting apoptosis as a strategy for cancer drug discovery. Nat Rev Cancer. 2005;5(11):876–885. 10.1038/nrc1736 16239906

[57] Hanahan D, Weinberg RA. The Hallmarks of cancer. Cell. 2000;100(1):57–70. 10647931 10.1016/s0092-8674(00)81683-9

[58] Chinni SR, Li Y, Upadhyay S, Koppolu PK, Sarkar FH. Indole-3-carbinol (I3C) induced cell growth inhibition, G1 cell cycle arrest and apoptosis in prostate cancer cells. Oncogene. 2001;20(23):2927–2936. 10.1038/sj.onc.1204365 11420705

[59] Giannakakou P, Robey R, Fojo T, Blagosklonny MV. Low concentrations of paclitaxel induce cell type-dependent p53, p21 and G1/G2 arrest instead of mitotic arrest: molecular determinants of paclitaxel-induced cytotoxicity. Oncogene. 2001;20(29):3806–3813. 10.1038/sj.onc.1204487 11439344

[60] Nakano K, Vousden KH. PUMA, a novel proapoptotic gene, is induced by p53. Mol Cell. 2001;7(3):683–694. 11463392 10.1016/s1097-2765(01)00214-3

[61] Yu J, Zhang L. PUMA, a potent killer with or without p53. Oncogene. 2008;27(1):S71–83. 19641508 10.1038/onc.2009.45 PMC2860432

[62] Kumari SR, Mendoza-Alvarez H, Alvarez-Gonzalez R. Functional interactions of p53 with poly(ADP-ribose) polymerase (PARP) during apoptosis following DNA damage: covalent poly(ADP-ribosyl)ation of p53 by exogenous PARP and noncovalent binding of p53 to the M(r) 85,000 proteolytic fragment. Cancer Res. 1998;58(22):5075–5080. 9823314

[63] Borner C. The Bcl-2 protein family: sensors and checkpoints for life-ordeath decisions. Mol Immunol. 2003;39(11):615–647. 12493639 10.1016/s0161-5890(02)00252-3

